# Using systematized tacit knowledge to prioritize implementation challenges in existing maternal health programs: implications for the post MDG era

**DOI:** 10.1093/heapol/czw033

**Published:** 2016-04-09

**Authors:** Victor Becerril-Montekio, Jacqueline Alcalde-Rabanal, Blair G Darney, Emanuel Orozco-Nuñez

**Affiliations:** ^1^Instituto Nacional de Salud Pública / Centro de Investigación en Sistemas de Salud (National Institute of Public Health / Centre for Health Systems Research), Av. Universidad 655, Col. Santa María Ahuacatitlán, Cuernavaca, Morelos CP 62100, Mexico; ^2^Department of Obstetrics and Gynecology, Oregon Health and Science University, 3181 SW Sam Jackson Park Rd, Portland, OR 97239, USA

**Keywords:** : Communities of practice, implementation research, low- and middle-income countries, quality, maternal health services, maternal mortality, tacit knowledge

## Abstract

Strategic priority setting and implementation of strategies to reduce maternal mortality are key to the post Millennium Development Goal (MDG) 2015 agenda. This article highlights the feasibility and the advantages of using a systematized tacit knowledge approach, using data from maternal health program personnel, to identify local challenges to implementing policies and programs to inform the post MDG era. Communities of practice, conceived as groups of people sharing professional interests, experiences and knowledge, were formed with diverse health personnel implementing maternal health programs in Mexico and Nicaragua. Participants attended several workshops and developed different online activities aiming to strengthen their capacities to acquire, analyze, adapt and apply research results and to systematize their experience and knowledge of the actual implementation of these programs. Concept mapping, a general method designed to organize and depict the ideas of a group on a particular topic, was used to manage, discuss and systematize their tacit knowledge about implementation problems of the programs they work in. Using a special online concept mapping platform, participants prioritized implementation problems by sorting them in conceptual clusters and rating their importance and feasibility of solution. Two hundred and thirty-one participants from three communities of practice in each country registered on the online concept mapping platform and 200 people satisfactorily completed the sorting and rating activities. Participants further discussed these results to prioritize the implementation problems of maternal health programs. Our main finding was a great similarity between the Mexican and the Nicaraguan general results highlighting the importance and the feasibility of solution of implementation problems related to the quality of healthcare. The use of rigorously organized tacit knowledge of health personnel proved to be a feasible and useful tool for prioritization to inform implementation priorities in the post MDG agenda.

Key Messages

- The use of rigorously organized tacit knowledge of health personnel proves to be a powerful tool for prioritization in maternal health programs.
- We report on an innovative approach to using tacit knowledge from health personnel on the front lines of care to develop priorities for quality improvement activities or implementation research.
- It is pertinent and useful to use health personnel’s tacit knowledge to set post MDG agendas at the local level.
- According to this tacit knowledge and its support on scientific literature, improving the quality of care in maternal health programs should be in the first place to guide post MDG agendas.

## Introduction

Maternal mortality remains a priority global health concern, and health programs addressing it face great expectations to achieve stated health and policy goals. Despite a number of policies and programs targeting Millennium Development Goal (MDG) 5 (to reduce maternal mortality by 75% between 1990 and 2015), only a few countries will reach this goal. Two years before 2015, the global average reduction only reached 45% (United Nations 2014). In 2013, the maternal mortality ratio in developing regions (230 maternal deaths per 100 000 live births) was 14 times higher than that of developed regions (United Nations 2014). Even though important progress has been made in almost all regions, many countries, among them Mexico and Nicaragua, will not meet MDG5. Failure to achieve desired results has been explained as failure of implementation, particularly in developing countries (Peters *et al.* 2013).

In low- and middle-income countries (LMICs), although the design of policies and programs is increasingly based on available scientific evidence, it is not yet very common to find evidence-based decision making about the implementation of public health interventions (González-Block *et al.* 2008; 2011). The implementation of maternal health programs encompasses an array of real-world actors and processes involving health personnel, the adequate use of resources and the delivery of services, at the right time and for properly identified health needs. Thus, successful implementation of evidence-based interventions requires both organizational decisions at different levels to adopt an intervention and its actual implementation or everyday functioning, all of which will determine failure or success (González Block et al. 2011).

The need to develop reasonable and equitable priority setting mechanisms concerning maternal health is highly recognized (Shiffman 2007). Priority setting becomes particularly important in contexts where resources are limited. Different approaches to priority setting, mainly economical, have been advanced (Nair *et al.* 2014; Stefanini 1999). Notwithstanding these elements, the kind of evidence to be considered in public health should include not only the different perspectives of scientific research but also other factors such as the knowledge and views of the diverse stakeholders participating in maternal health programs (Nair *et al.* 2014; Panisset *et al.* 2012).

This article uses data from an Alliance for Health Policy and Systems Research (AHPSR)/World Health Organization-funded project that tested a capacity strengthening model for maternal health personnel in two LMICs: Mexico and Nicaragua. The project goal was based on existing evidence and aimed to strengthen health personnel’s capacity to use research results and to understand the core focus and characteristics of implementation research to guide evidence-based decision making in maternal health programs (González-Block *et al.* 2011; Rouvier *et al.* 2013).

Existing maternal health programs in Mexico and Nicaragua promote scientifically proven interventions; therefore, the capacity-building model of this project was initially oriented towards the utilization of implementation research (IR), the branch of health systems research dedicated to study the way any health intervention is made available to the public, answering how and why scientifically proven interventions fail or succeed in reaching their goals (Peters *et al.* 2013). Moreover, as Peters *et al.* (2013 p. 9) state, ‘implementation research is often at its most useful where implementers have played a part in the identification, design and conduct phases of the research undertaken’, combining the objective of strengthening maternal health programs’ personnel to use and apply scientific evidence with this characteristic of IR, the project worked on the systematization of the knowledge and perspectives of the people actually working in them. Because the actual implementation of policies and programs in local settings is always related to contextual factors, we considered the role of tacit knowledge as an important element that can offer important information on how national policies and programs developed to achieve the MDGs are implemented and function locally.

We considered the tacit knowledge of the people working in the implementation of maternal health programs as a potentially useful approach to identify and prioritize implementation challenges that need to be addressed. Tacit knowledge has been defined as a kind of knowledge that every human being has a relation to his or her everyday practice of a task or profession (Business Dictionary 2014; Polanyi 1966). Tacit knowledge is always linked to a particular individual, making it hard to formalize and communicate (Nonaka 1994). It includes two dimensions: (1) a technical element that has been described as the *know how* related to certain skills in a particular context and (b) a cognitive element that ‘refers to the individual’s images of reality and visions for the future, that is to say, what is and what ought to be’ (Nonaka 1994, p. 16). Other authors also note that it is usually shared through informal channels, and that to be transferred, it requires close or extensive personal contact and trust (Ambrosini and Bowman 2001; Kothari *et al.* 2011; Landry *et al.* 2006; McAdam *et al.* 2011). Finally, to assure its best utilization, tacit knowledge needs to be socialized and systematized.

The purpose of this article is to assess the feasibility of using the tacit knowledge methodology to prioritize challenges to implementation of current maternal health programs and inform the post MDG agenda in LMICs.

## Methods

In this project, we implemented a participative approach by creating six communities of practice (CoPs). CoPs function as interactive interfaces in which researchers and practitioners can meet, motivated by common interests while they also foster their members’ development both personally and professionally (Bertone *et al.* 2013, Best *et al.* 2007; Wenger 1998). The CoPs were formed in three states of Mexico and three departments of Nicaragua and selected using three basic criteria: (1) relatively high (above national averages) rates of maternal mortality; (2) similar human development indexes and (3) presence of health authorities willing to participate in the project. The project included the states of Hidalgo, Morelos and Veracruz in Mexico and the departments of Chontales, Jinotega and Matagalpa in Nicaragua. The CoPs were formed during May to June 2013 following a call for participation launched by the health authorities in each state or department. Participants included managers of maternal health programs and personnel in contact with patients and other healthcare providers or administrators (Table 1). Participation in these CoPs was voluntary. In agreement with the corresponding health authorities, a person with expertise in information and communication technologies was named as a facilitator to organize each CoP and serve as link with the research team. We sought to engage the CoPs in a priority-setting process based on their tacit knowledge of implementation challenges, face-to-face workshops and a participatory online forum for the concept mapping exercise. These activities were held from July to December 2013.

**Table 1.** Socio-demographic composition of CoP members from Mexico and Nicaragua with useful answers in Concept Mapping activities

Variables	Mexico		Nicaragua		Total	
	Total	%	Total	%	Total	%
Sex						
Male	26	32.1	37	31.09	63	31.5
Female	55	67.9	82	68.91	137	68.5
Age						
<30	13	16.05	52	43.7	65	32.5
31–50	63	77.78	58	48.74	121	60.5
>50	5	6.17	9	7.56	14	7
Education^a^						
Below University	9	11.11	41	34.45	50	25
University/nursing/physician	51	62.96	62	52.1	113	56.5
Postgraduate	21	25.92	16	13.44	37	18.5
Nurse	26	32.09	50	42.02	76	38
Profession						
Physician	38	46.91	21	17.65	59	29.5
Manager	6	7.41	19	15.97	25	12.5
Other	11	13.58	29	24.37	40	20
Place of work						
Primary care	53	65.43	77	64.71	130	65
Hospitals	17	20.99	23	19.33	40	20
Administration	11	13.58	19	15.97	30	15
Total	81	40.5	119	59.5	200	100

* Note In Mexico and Nicaragua physicians have a university level degree, while nurses may or may not have it.

Concept mapping was our main tool to extract and systematize what we defined as the ‘raw’ tacit knowledge of each CoP about the implementation challenges of various maternal health programs. Concept mapping is a general methodology that helps to organize and graphically present the ideas of a group of people about a subject of common interest (Trochim 1989). All participants gave informed consent to participate in concept mapping, and the whole process was performed in the six CoPs of the two countries. The first workshop was based in the following protocol:

A brief didactic presentation on health systems, implementation and implementation research.
The concept mapping initial brainstorming session to answer the focus question: What are the main problems of the state or departmental health system that represent an obstacle to reach the expected results of maternal health programs?
Participants were organized in groups of 6–10 people and asked to individually write 5–15 answers to the focus question based on their own personal experience.
Individual answers to the question were recorded on cards.
After discussion, each group eliminated duplicated or redundant answers.

Using this material and a content analysis approach, the research team collated and synthesized answers from all CoPs eliminating duplicates and redundancies across groups and reducing the list to 98 statements (Hsieh and Shannon 2005). During a second workshop, participants were trained on the use of the Concept Systems Global platform, an online tool, to perform the rest of the concept mapping activities online (Concept Systems Global 2014). The research team uploaded the 98 statements to the platform, and the research team leader formatted the platform according to the specific objectives of the project.

Each CoP member was next asked to perform five tasks on the online platform:

Register on the platform creating their own usernames and passwords.
Provide basic anonymous sociodemographic information (country, state or department, age, gender, education and main responsibilities in the maternal health programs).
Sort the 98 statements in conceptual clusters (minimum of 5 and a maximum of 20) and name them according to their own criteria to finally save their personal sorting results.
Rate each statement according to its importance using a Likert scale in which 1 equals ‘not important’ and 5 equals ‘of vital importance’.
Rate the feasibility of solving the problem referred in each statement on a similar Likert scale in which 1 equals ‘cannot be solved’ and 5 equals ‘it’s already being solved’.

Facilitators of the CoPs were responsible for the follow-up with each member to assure timely and accurate participation. The research team reviewed and confirmed the validity of each member’s information.

Using the statements and the results of the online sorting exercise, we generated clusters or conceptual groups using multidimensional scaling and a correlation matrix embedded in the platform, then weighted each cluster using the priority and feasibility data provided by CoP members. The cluster or conceptual group names were discussed with the CoPs in order to make sure that they accurately represented the main ideas of the statements gathered in each cluster. We next tested varying numbers of clusters (8, 10 and 12 clusters in a map) and presented the options to the CoPs for discussion. Consensus was reached that 10 cluster maps were the best.

We next used the average values of the importance and feasibility of solution rating scores for each statement to generate a correlation to identify the importance–feasibility relationship at the statement level. Starting with the discussion on the clusters, the selection of the particular problems to be addressed through implementation research was made by each CoP after discussing on the problems combining the highest scores for both criteria (importance and feasibility). We thus finished with cluster-level and statement-level average ratings of importance and feasibility, as well as a measure of joint importance and feasibility.

A thorough analysis of the concept mapping results was made during a 40-h workshop and an additional training on evidence-based decision making in public health and implementation research with 25 leaders of the different CoPs. By the end of the week, each CoP had started developing an implementation research protocol focused on a priority implementation problem to be discussed and finalized with the participation of all of its members. The analysis of the concept maps also served as basis for the selection of problems upon which a more local action would be undertaken after the elaboration of small intervention protocols.

## Results

From the 298 participants in the brainstorming activities in both countries, 231 members of the six CoPs registered in the CSG platform and 200 people satisfactorily completed them (Table 1).

Our main finding was the similarity between the Mexican and the Nicaraguan general results as they appeared in their corresponding concept maps, particularly concerning both the importance and the feasibility of solution rating of two clusters: one containing the statements related to the *quality of healthcare* and another one gathering statements about the *lack of financial resources*. This led to consider the global concept maps of the two countries organizing the 98 ideas in 10 conceptual groups or clusters.

**Figure 1.**
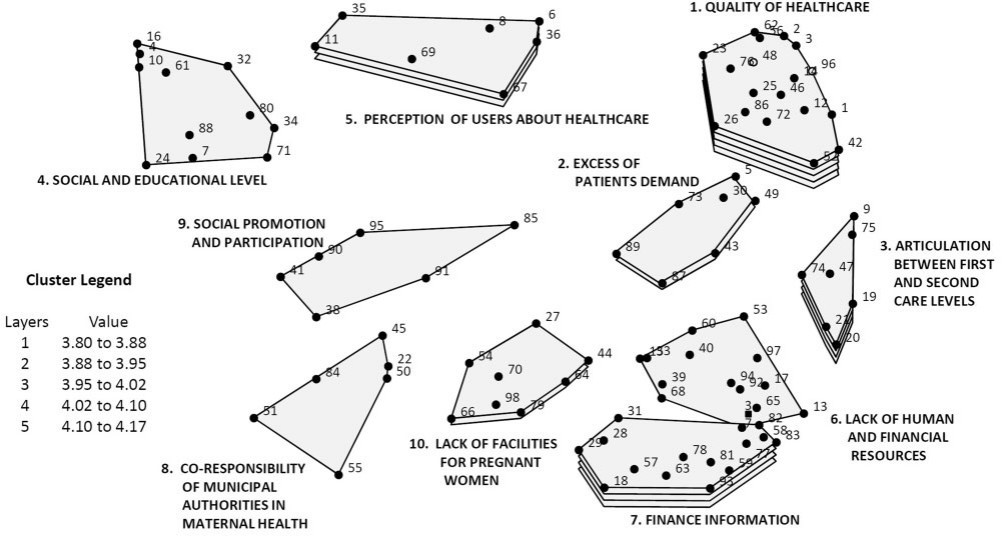
Concept map of systematized tactic knowledge on maternal health programs implementation problems. Averaging rating (importance), Mexico and Nicaragua

**Figure 2.**
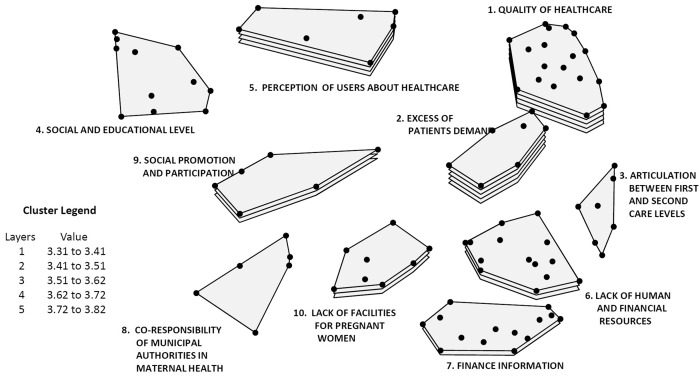
Concept map of systematized tactic knowledge on maternal health programs implementation problems. Averaging rating (feasibility), Mexico and Nicaragua

Figures 1 and 2 present the maps including the relevance of each cluster according to the average of its rating for importance (Figure 1) and feasibility of solution (Figure 2). The points in each cluster represent each one of the 98 problems identified by the CoPs, with their corresponding numbers, according to the random order given to them by the platform. The number of layers of each cluster represents the average rating: five layers represent the highest rating of the ideas, whereas only one layer is accorded to sets of ideas with the lowest rating average.

Cluster 1 ‘quality of healthcare’ in Figure 1 has five layers, showing the highest importance rating average, and it includes the largest number of statements (18 points), all of which describe different problems related to the quality of care. Two other clusters with four layers follow: Cluster 3 and Cluster 7. Including only two layers, Clusters 2 and 10 have average ratings between 3.88 and 3.95 while the average rating of the other three clusters with only one layer is below 3.88.

Concerning the feasibility of solution of the implementation problems, Figure 2 shows two clusters with high rating averages and, therefore, five layers: Cluster 1 ‘quality of healthcare’ and Cluster 3 ‘excess of patients demand’. The following cluster in feasibility of solution average rating is Cluster 5 with four layers, whereas three clusters show intermediate averages of feasibility rating: Clusters 6, 9 and 10. Instead, the rating of Cluster 7 corresponds to very difficult to resolve problems. Finally, the clusters that are the most difficult to resolve are 3 and 8.

**Figure 3.**
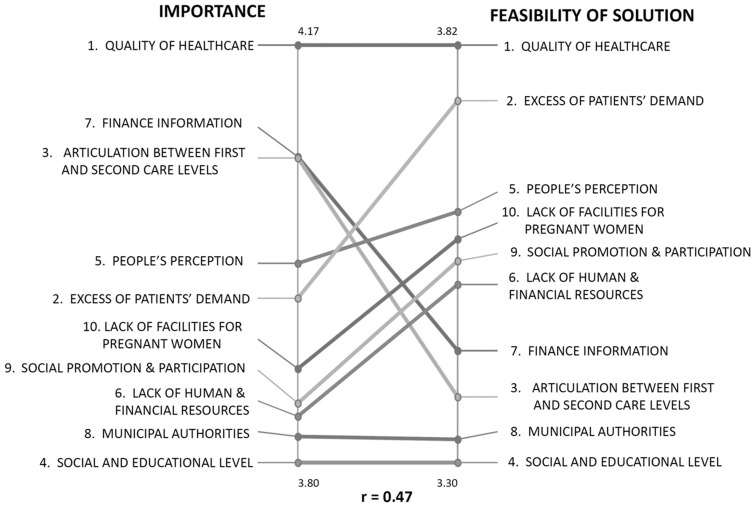
Correlation between average ratings of the 10 clusters of implementation problems of maternal health programs by Mexican and Nicaraguan CoPs

Figure 3 depicts the correlation of importance and feasibility ratings of each of the 10 clusters. This figure compares the average value of the two ratings of the statements included in each cluster. Some clusters were rated similarly—quality high on both, importance and feasibility, municipal authorities and social/educational level low on both—whereas others received different ratings for the two criteria.

**Figure 4.**
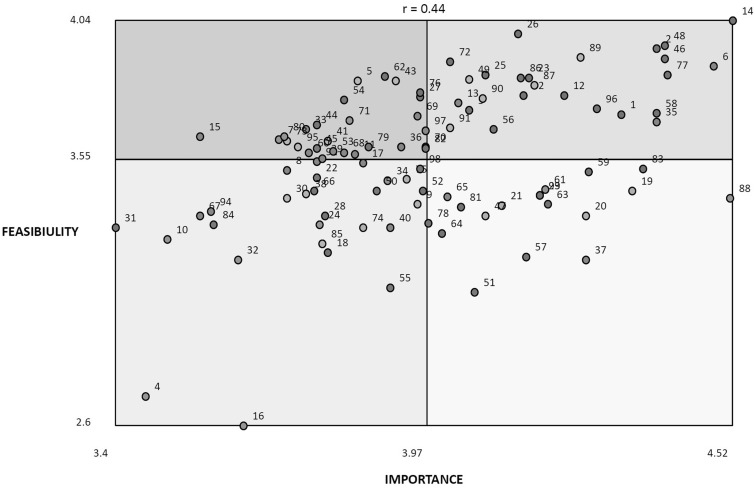
Strategic zones of average ratings of implementation problems of maternal health programs by Mexican and Nicaraguan CoPs

In addition to classifying the importance and feasibility rating of all clusters, we also classified individual statements according the highest average ratings for importance and feasibility of solution (Figure 4). The statements (problems) falling in Square I of Figure 4 combine the lowest rating for importance and feasibility of solution. Square II gathers those problems with a higher rating average for feasibility of solution, but a low rating concerning importance. Square III includes problems with a high importance average rating but low feasibility of solution. Finally, Square IV or Go Zone gathers those problems with the highest average ratings for importance and feasibility of solution. Logically, this is the priority zone where decision makers and researchers should look for the development of their proposals. The most important finding is that 15 out of 25 problems in Square IV focus on quality issues. Although the clusters correlation permitted to give a general orientation towards quality of health care, the position of each statement in Figure 4 permitted to focus, in particular, on implementation problems.

Table 2 also shows that out of 25 statements in the Go Zone, 15 are part of Cluster 1 ‘quality of healthcare’, whereas the other 10 are distributed among five other clusters.

**Table 2.** Individual statements that rated high on importance and feasibility, Mexican and Nicaraguan CoPs

Statement Number	Statements rated high on both importance and feasibility Green Go Zone	Included in Cluster
1	Consultations for risk pregnancies are established in distant dates and even after the delivery date	Quality of care
2	Deficient valuation of pregnant women by the personnel who receives them (physicians and nurses)	Quality of care
3	Negative attitude during care of personnel towards pregnant women	Quality of care
6	Pregnant women and their families fail to make a timely detection of alarm signs	People’s perception
12	Bad reception of pregnant women in emergency cases by surveillance personnel in health units	Quality of care
13	Problems with the distribution of delivery care inputs	Lack of resources
14	Deficient quality of care during pregnancy, delivery and postpartum	Quality of care
23	High rate of refusal of care for pregnant women in health units	Quality of care
25	Lack of follow-up of postpartum by health care personnel	Quality of care
26	Lack of compliance to Official Norms and Practice Guides	Quality of care
35	Pregnant women fail to attend to antenatal control	People’s perception
42	Long waiting time for pregnant women’s care in health units	Quality of care
46	Inadequate follow-up of obstetric complications	Quality of care
48	Inadequate identification of obstetric risk by health personnel	Quality of care
49	Lack of follow-up and evaluation of maternal health care processes	Excess of demand
56	Lack of human attitudes in health personnel responsible for pregnant women’s care	Quality of care
58	Obstetric emergency and general care equipment is in bad conditions	Finance information
72	Limited follow-up of pregnant women in the first level of care	Quality of care
77	Lack of drugs for normal and emergency obstetric care	Finance information
86	Lack of follow-up in pregnant women’s care to guarantee an integral care	Quality of care
87	Human resources for health are badly trained during their studies	Excess of demand
89	Health personnel lacks training	Excess of demand
90	Sexual education programs for adolescents are not implemented	Social promotion
91	Community personnel linked with maternal health programs lacks training	Social promotion
96	Negligence on the part of health personnel	Quality of care

Elaborated from the CSG platform analysis of statements.

After analyzing their own systematized tacit knowledge, the leaders of each CoP focused on the priority clusters and individual statements rated highly on both importance and feasibility of their state or department and decided upon the implementation problems they would choose either to develop a small local intervention protocol or a larger implementation research protocol. The first aimed at improving the implementation of local issues with high and relatively simple feasibility of solution concerning such things as prenatal control, obstetric risk identification, health professionals training, post-natal follow-up and the referral system. In the cases of problems demanding a deeper consideration and study, implementation research protocols were designed.

For the implementation research protocols, in Mexico, the CoP Hidalgo chose statements 2 (‘deficient valuation of pregnant women by the personnel who receives them -physicians and nurses’) and 14 (‘deficient quality of care during pregnancy, delivery and postpartum’). The choice of the CoP from Morelos was statement number 6 (‘pregnant women and their families fail to make a timely detection of alarm signs’), also in the Go Zone of both countries. In the case of Veracruz, the CoP also selected statement 14, but did not link it to any other one. In Nicaragua, also finding a relevant relationship between statements 14 and 26 (‘lack of compliance to official norms and practice guides’), the cops of Chontales and Jinotega resolved to address the quality of care *vis-à-vis* the Health Ministry’s norm on prenatal care (Ministerio de Salud 2008). Finally, the CoP of Matagalpa focused on statement number 48 (‘inadequate identification of obstetric risk by health personnel’) to work on an IR protocol.

## Discussion

We report on an innovative approach to using tacit knowledge from health personnel on the front lines of care to develop priorities for quality improvement activities or implementation research. Our Communities of Practice and concept mapping approach was feasible and acceptable to participants. Tacit knowledge is supported by what we know from the literature. It is feasible and valuable to use tacit knowledge to prioritize problems in the implementation of maternal health programs that can eventually inform the post-MDG agenda at the local level. We are aware that tacit knowledge might leave certain issues aside, as could be the case of unsafe abortion, HIV and other stigmatized issues. So, even though we found it useful, we recognize that the tacit knowledge approach needs to be complemented by other methods. Our key finding is that deficiencies in the quality of maternal health care emerged as a priority issue based on the systemized tacit knowledge of participating healthcare personnel in both Mexico and Nicaragua. There was clear agreement on the high importance of problems related to the quality of care and on the high feasibility of addressing such problems. When compared with the other nine conceptual groups, the quality of care cluster, not only combines the highest rating for importance and feasibility of solution but also gathers the largest number of statements (18 points). Finally, 15 out of 25 individual statements describing implementation problems with the highest rating average for both criteria are quality of care problems.

CoPs in both countries identified factors determining deficient quality of care in public healthcare facilities that include pregnancy, childbirth and post-partum (Yemile Ordaz-Martínez *et al.* 2010). They considered human resources problems such as the lack of them or their deficient training, particularly in first-level health facilities (McIntosh *et al.* 2012). The absence of the humane sensibility among health personnel was also identified, as was the inaccurate assessment of obstetric risk, from which stems a deficient follow-up. Due to problems in timely admission, pregnant women are forced to look for care elsewhere and many complications derive from the delays originated in bad reference and counter-reference (Collado-Peña and Sánchez-Bringas 2012; Lassi *et al.* 2014). Another aspect of the quality of healthcare that was recognized was the refusal of obstetric healthcare that women sometimes suffer in public facilities. These practices highlight the bad quality of care and the violation of the right to receive healthcare (Pérez-Castro y Vázquez *et al.* 2012). CoPs also recognized a persistent lack of drugs and other inputs, which specially hinders the adequate care of obstetric emergencies (Rouvier *et al.* 2011). Finally, different problems limit the proper follow-up of post-natal care, a period during which many obstetric complications might arise causing maternal deaths (Pérez-Castro y Vázquez *et al.* 2012).

The organization and delivery of maternal health services must be able to address unpredictable events and high-risk situations (DuPree *et al.* 2009; Institute of Medicine 2001). One tool to foster best practices, monitor and improve quality in direct clinical encounters is the World Health Organization Childbirth Checklist Programme (World Health Organization 2013). But there is also a need to interventions to improve quality at the health system level (Huntington *et al.* 2012). Strategies for improving the quality of maternal health care in LMICs, such as educational programs for women, capacity strengthening programs for health professionals, integration of services, use of clinical practice guidelines, provider incentives and other related to the support of local opinion leaders have been identified (Althabe *et al.* 2008).

Notwithstanding the important progress made during the past decades in public hospitals of Mexico and Nicaragua, both countries still face a long agenda to reach good quality standards in maternal healthcare programs, particularly in rural areas (Barber *et al.* 2007). The prioritization made by the CoPs is based on the experience of people directly responsible of the administration or the health care provided by maternal healthcare programs. And it is not a matter of mere coincidence that they recognize bad quality of public healthcare services. The results of their systematized tacit knowledge find substantial support in a large spectrum of scientific evidence and represent a feasible approach that can inform priority setting in the post MDG era (Nair *et al.* 2014).

## Conclusions

The problems that were identified and prioritized based on the tacit knowledge of health system actors are supported by scientific literature. We therefore consider tacit knowledge is a feasible and potentially valuable approach to inform the post MDG agenda at the local level. Tacit knowledge, as it is initially externalized by the health personnel, can be considered as some kind of ‘raw’ material that has to be managed using scientifically sound methods. There is certainly not one single way to do this. Nevertheless, it is essential to assure its systematization. The experience presented here was essentially based on concept mapping combining face-to-face and virtual exchanges among participants. No doubt researchers and health workers can find other ways to make the best use of the latters’ tacit knowledge.

Improving the quality of care in maternal health programs becomes the *sine qua non* strategy to assure universal and effective coverage and stands in the first place to guide the post MDG agendas. Based on this new perspective, strategic planning should aim to work on building the necessary capacities of the health system and the health personnel in care provision and its management.
